# Theoretical Analysis of a Nonequilibrium Transport Model of Two-Dimensional Nonisothermal Reactive Chromatography Accounting for Bi-Langmuir Isotherm

**DOI:** 10.1021/acsomega.2c06317

**Published:** 2023-01-12

**Authors:** Sadia Perveen, Muhammad Afraz Rasheed, Samra Sana, Iram Mumtaz, Shamsul Qamar

**Affiliations:** † Department of Mathematics, Air University, Islamabad, 44000, Pakistan; ‡ Department of Mathematics, COMSATS University Islamabad, Islamabad, 45550, Pakistan

## Abstract

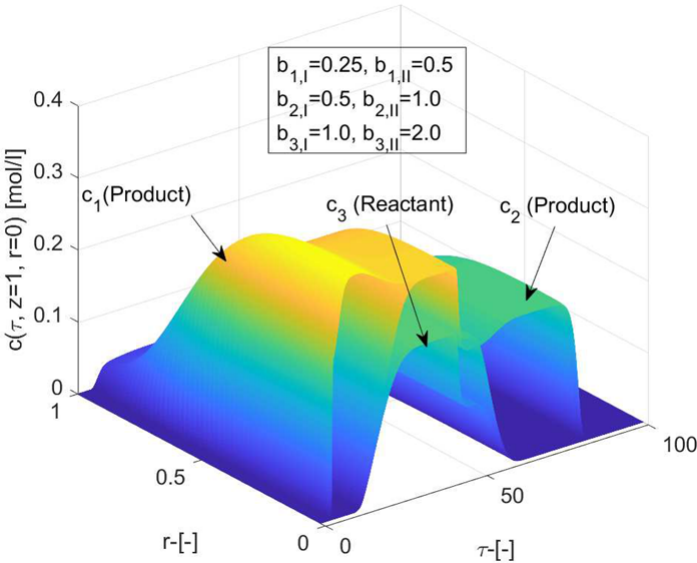

The current study investigates a nonequilibrium and nonlinear two-dimensional lumped kinetic transport model of nonisothermal reactive liquid chromatography, considering the Bi-Langmuir adsorption isotherm, heterogeneous reaction rates, radial and axial concentration variations, and the adsorption and reaction enthalpies. The mathematical models of packed bed chromatographic processes are expressed by a highly nonlinear system of coupled partial differential algebraic equations connecting the phenomena of convection, diffusion, and reaction, for mass and energy balance, the differential algebraic equations for mass balance in the solid phase, and the algebraical expressions for the adsorption isotherms and for the reaction rates. The nonlinearity of the reaction term and the adsorption isotherm preclude the derivation of an analytical solution for the model equations. For this reason, a semidiscrete, high-resolution, finite-volume technique is extended and employed in this study to obtain the numerical solution. Several consistency checks are performed to evaluate the model predictions and analyze the precision of the proposed numerical scheme. A number of heterogeneously catalyzed stoichiometric reactions are numerically simulated to examine reactor performance under the influence of temperature and Bi-Langmuir adsorption dynamics, the level of coupling between mass and energy fronts, and to study the effects of various critical parameters. The numerical results obtained are beneficial for optimal predictive control and process optimization during production and the development of methods for systematic design and fault detection of nonisothermal liquid chromatographic reactors, and hence constitute the first step to provide deeper insight into the overall evaluation of integrated reaction and separation processes.

## Introduction

The components of a mixture are not chemically bonded together; thus, some physical mechanism can be utilized for their separation. The physical separation techniques like evaporation, filtration, distillation, and chromatography use differences in the physical properties to isolate the components of a mixture. Among the aforementioned techniques, chromatography is an efficient, accurate, and effective technique used in industries and laboratories to separate and analyze different kinds of complex chemical mixtures. The ongoing progress in science and technology has further revolutionized this process.^1^ This technique can be effectively applied to targeted separation tasks for producing high-purity products at economical production rates. Chromatography is frequently applied as a separation and purification technique in fine chemical, petrochemical, biotechnical, pharmaceutical, forensic pathology, nucleic acid research, and many more areas.^2^

The development of sustainable chemical processes is a crucial issue in the modern world. The chemical processes are generally made up of reactors, separation and recycle systems, mixing, changes in pressure, changes in particle size, heating or cooling utilities, heat recovery systems, and the water and effluent treatment systems. The synthesis of a reactive chemical network involves two main activities. The first activity includes the selection of individual transformation steps. In the second activity, to construct a complete process that carries out the desired overall transformation, these individual transformations must be interconnected.^3^ A numerical simulation for the mathematical model of the process can then be carried out to predict the compositions, flow-rates, temperatures, and pressures of the products. The primary performance of the design can then be evaluated, and further changes can be made to improve performance. The process is then optimized.

Integration of a chemical reaction and separation in one single processing unit has acquired considerable importance in the recent past. The chemical process industries have recognized this combination as having affirmative economics for executing reactions simultaneously with separation for specific types of reacting systems, and several new integrated procedures have been developed on the basis of this technology. In contrast to conventional reactors and separators that are sequentially connected, this attractive technique has been found capable of separating complex mixtures with improved conversion, yield, and separation capacity.^4−7^ The main objective of the method is to increase productivity, enhance selectivity, eliminate the need for solvents, reduce energy use, and thus lead to highly efficient intensified systems. Because of the dynamic nature of this multifunctional reactive process, heat is continuously produced and consumed due to the adsorption enthalpy, mixing, and chemical reaction. Moreover, thermal effects may also result from the dissipation of viscous heat, especially in fine particulate high performance liquid chromatographic columns.^8,9^ Such phenomena should be considered when scaling the reactor perception, as increasing the column diameter ultimately makes the system almost adiabatic.

A chemical reaction within the catalytic reactor can be classified according to its phase conditions as a homogeneously or heterogeneously catalyzed reaction. Homogeneous catalysis refers to reactions where the catalyst and the reactants are in the same phase, principally the liquid mobile phase. Thus, the separation of the product from the catalyst has to be considered at the end of the process. On the contrary, in heterogeneous catalysis, the reactants adsorb onto binding sites on the catalyst surface, and the availability of these reaction sites can limit the rate of heterogeneous reactions. The reactive chromatography has a wide range of applications like esterification,^10^ etherification^11^ acetalization,^12,13^ transesterification,^14^ alkylations,^15^ reactions involving sugar,^16,17^ and hydrolysis^18,19^ as well as (de)hydrogenation.^20^

The nature of the reaction and the component’s elution order significantly influence the reactive chromatographic procedures. Mainly, the equilibrium limited reaction of the type *R* ⇄ *P*, *R* ⇄ *P*_1_ + *P*_2_, and *R*_1_ + *R*_2_ ⇄ *P*_1_ + *P*_2_ are thoroughly investigated in the literary texts for isothermal and nonisothermal cases considering linear and nonlinear adsorption isotherm.^21−32^ Continuous separation of at least one of the reaction products shifts the equilibrium in a direction that enhances the conversion rate and limits byproduct formation in equilibrium limited reactions. Batch mode is the simplest way to operate a chromatographic reactor. The batch reactive chromatography principle can be easily explained using a reversible chemical reaction *R* ⇄ *P*_1_ + *P*_2_. A reactive desorbent is fed as a rectangular pulse into the reactor, which is loaded with a solid adsorbent with catalytic properties. The reaction takes place at the surface of the catalyst and forms the products *P*_1_ and *P*_2_. The two products interact with the adsorbent’s surface and will move along the column with different propagation velocities owing to their individual affinities with the solid bed. The products are collected separately by fractionation on account of their different elution times.

High-temperature liquid chromatography (HTLC) has piqued the interest of many in recent years, but its full potential in the chromatographic community has yet to be realized. The industry has a general reluctance to employ temperature to accelerate the separation process, influence the separation selectivity, or implement innovative detection techniques.^33^ Column temperature is eminently important in the formation, growth enhancement, and optimization of high performance liquid chromatography (HPLC).^34−37^ Temperature variations, which can be introduced internally or externally, are primarily used in liquid chromatography to manipulate the transportation speeds of the components within the column for improving separation or column efficiency. In HTLC, mass transfer kinetics, diffusion characteristics, and separation processes can be improved due to their dependency on temperature. Temperature fluctuations can improve column performance by abbreviating analysis and separation times, reducing the consumption of organic solvents, sharpening elution profiles, and speeding up the conversion of reactants into products in applications of reactive chromatography.^38,39^ High-temperature liquid chromatography (HTLC) significantly improves HPLC’s functionality in handling complex samples. To ensure the quality of HTLC separation and analysis, the stationary phases should remain stable under high-temperature operation. HTLC separations for thousands of column volumes can be performed using newly developed stationary phases that are thermally more stable than traditional bonded silica. Commercial column heaters are now available that allow operation up to 200 °C with mobile phase preheating, reducing the negative effects of thermal inconsistency.^40,41^ Nonisothermal chromatography has been studied extensively in the literature to illustrate thermal effects on elution profile retention behaviors, concentration variations, packing materials, the injected pulse volumes, and also on the ion-exchange chromatography.^42−45^

The hydrodynamic behavior of internals influences integrated separation and reaction processes in addition to complex multicomponent thermodynamic behavior and simultaneous chemical reactions. To adequately describe these phenomena, sophisticated mathematical models that encompass mass transfer phenomena, fluid dynamics, and multifaceted chemical reaction schemes have been developed for estimating model parameters from experimental data and are highly beneficial in the realm of chemical engineering. It is a firmly entrenched mechanism to employ digital technology for simulating reactive chromatographic operations on an industrial scale. It is essential for optimizing, developing, designing, and interpreting many chemical engineering processes. The modeling of chromatographic procedures provides an indispensable framework for predicting and interpreting the fluid transport phenomena in the column rather than using conventional and time-consuming experimental methods. Due to its computational accuracy and performance, numerical simulation is a valuable technique for dealing with complicated problems.^46,47^ Several dynamical models, taking into consideration diverse levels of complexity, have been introduced to evaluate the principal performance of the chromatographic reactors. The selection of a suitable model is based on the specific modeling design objectives. The ideal model (IM), the equilibrium dispersive model (EDM), the nonequilibrium transport models, for example (LKM & LPDM), the equivalence of the macroscopic kinetic model (EMKM), and the general rate model (GRM) are the prevalent chromatographic models.^48−51^ The linearity and nonlinearity of these models depend on the adsorption isotherms associated with them. In the literature, several shock-capturing, high order numerical techniques in accordance with the total variation diminishing framework are commonly used for cost-effective simulation of nonlinear chromatographic models. These numerical techniques acquire stable solution profiles with high order accuracy in the smooth region and without introducing oscillations near discontinuities. The nonoscillatory finite difference (FD) methods like total variation diminishing (TVD), total variation bounded (TVB), weighted essentially nonoscillatory (WENO), essentially nonoscillatory (ENO), the flux limiting finite volume methods (FVMs), and the discontinuous Galerkin finite element method (DG-FEM) are among the few numerical methods with the ability to resolve discontinuities in the solution profiles.^52−61^

In the present study, we formulate and simulate numerically a nonequilibrium 2D-RLKM to theoretically investigate the functioning of chromatographic reactive processes operating under nonisothermal, nonlinear adsorption conditions characterized by Bi-Langmuir adsorption isotherm.^62,63^ This study dilates and continues our recent theoretical investigation of nonreactive, isothermal liquid chromatography by incorporating a two-dimensional lumped kinetic model (2D-LKM).^64^ A reversible reaction of the form *R* ⇄ *P*_1_ + *P*_2_ is considered in the current study to theoretically investigate, reaction-separation kinetics and adsorption equilibria in a (2D) batch chromatographic reactor. The considered 2D-RLKM is based on a coupled system of nonlinear partial differential-algebraic equations (PDAEs). The stratagem for the numerical solution of the complex nonlinear model equations is contingent on a precise and efficient HR-FVM.^65^ HR-FVM is used to discretize the axial and radial coordinates, while the time derivative remains constant. Then, the resulting system of ODEs is solved by utilizing the second-order accurate Runge–Kutta approach. The main objectives of this research work include (i) the investigation of a nonequilibrium and nonisothermal, 2D reactive liquid chromatography process assuming double adsorption sites (i.e., the Bi-Langmuir adsorption isotherm), and (ii) the formulation of the HR-FVM method for the numerical solution of 2D-RLKM. Numerous case studies of practical relevance are conducted to study the coupling between thermal and concentration fronts in the reaction-separation process. As chromatographic reactors still lack experimental evidence for validating their mathematical models. Therefore, integral-consistency analysis is introduced in this study for verifying the validity and accuracy of the applied numerical scheme. Moreover, key parameters have been identified that influence reactor performance.

The remaining article is structured as follows. The 2D-RLKM for a nonisothermal reactive liquid chromatography considering Bi-Langmuir adsorption is formulated in the section “Formulation of Nonisothermal 2D-RLKM”. The section “Simplification of Mathematical Model” introduces dimensionless quantities to further simplify the mathematical model along with the description of initial and boundary conditions. The section entitled “Implementation of HR-FVM” refers the readers to Appendix I “Derivation of Numerical Scheme” for the derivation of the high resolution finite-volume technique (HR-FVM) for the model equations. The technique of consistency checking to validate the results of the proposed numerical scheme is summarized in the section “Validation of Numerical Results through Consistency Test”. In the section “Results and Discussion on Numerical Case Studies,” the impact of various thermodynamic and kinetic parameters on the efficacy of the 2D chromatographic reactor is demonstrated. In addition, the study concludes with a section titled “Conclusion.”

## Formulation of Nonisothermal 2D-RLKM

**Figure 1:**
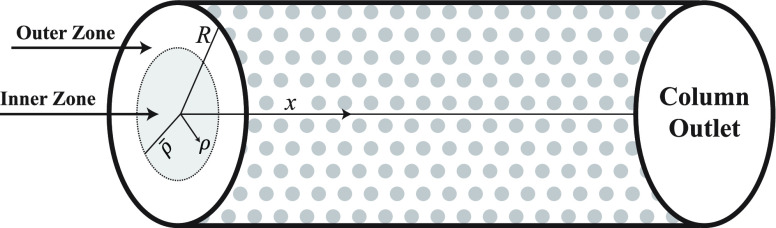
Process diagram of thermally insulated chromatographic reactor illustrating reactant injection through two distinct cylindrical zones.

The nonequilibrium transport of products and reactants in a two-dimensional, thermally insulated chromatographic reactor replete with porous particles is considered in the present research. The model considers a heterogeneously catalyzed solid-phase reversible reactions of the type *c*_3_ ⇄ *c*_1_ + *c*_2_. The adsorption column reactor incorporates axial and radial dispersion, mass and heat transfer impedance, and Bi-Langmuir adsorption kinetics. A sharp pulse of reactant (*c*_3_) is injected into the concentration of adsorbate at initial time. The products (*c*_1_ and *c*_2_) are produced by the continuous decay of the reactant via a heterogeneous reaction. The reactant and products migrate by convection and axial dispersion in the *x*-direction along the reactor axis and disseminate radially by radial dispersion in the ρ-direction along the reactor radius. To escalate the effects of mass and heat transfer along the radial direction, the inlet cross sectional area of the cylindrical reactor is bifurcated into two regions through the introduction of a new parameter denoted by the symbol ρ̅ (see Figure 1): (a) the annular outer-ring; (b) the cylindrical inner-core. Consequently, there will be three distinct possibilities for injecting the reactant in the chromatographic reactor. The reactant can be injected either over the cylindrical inner core, via an annular outer ring, or over the whole column reactor cross section. It is noteworthy that these injection modes have a similarity to the annular chromatographic procedures whereupon the column reactor rotates.^66^ The model formulation is based on the following assumptions:

(i) The adsorbent bed is heterogeneous and is concealed by two absolutely autonomous adsorption sites.
(ii) The chromatographic reactor is uniformly replete with spherical porous particles of radius *R*_*p*_.
(iii) The dispersion and the thermal conductivity coefficients along the spatial directions are taken independent of the flow rate.
(iv) The viscous heat of the system and flow rate fluctuations are negated.
(v) The mobile phase is incompressible and there is no interactivity between the solvent and the solid phase.
(vi) Physical properties of the fluid such as heat capacity, density, viscosity, transport coefficients such as heat conductivities, and dispersion of space variables are considered to be temperature independent.
(vii) It is assumed that no heat is interleaved or released by the reactor walls except for the inlet or outlet streams.
(viii) The overall adsorption rate is described using a solid film linear driving force model.

In reference to the above assumptions, the primary material balance equations for the conservation of mass and energy of a two-dimensional multicomponent, nonisothermal, 2D-RLKM are given as^49^
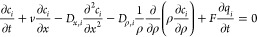
1
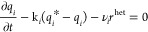
2
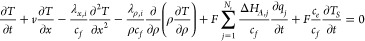
3
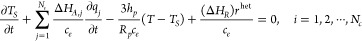
4Here, the coordinates, *t*, *x*, and ρ denote the time, the axial position, and the radial position along the column. For the *i*^th^ component of the solute, *c*_*i*_(*t*, *x*, ρ) and *q*_*i*_(*t*, *x*, ρ) signified the concentrations in the mobile and solid phases, respectively, *v* denotes the constant liquid phase velocity, and *D*_*x*,*i*_ and *D*_ρ,*i*_ are the dispersion coefficients of the space variables *x* and ρ. The phase volume ratio is with ϵ_*c*_ being the external porosity, *q*_i_^*^ is the temperature dependent equilibrium solid phase concentration, k_*i*_ is the coefficient of mass transfer rate, ν_*i*_ is the stoichiometric coefficient of the *i*^th^ component of the sample, and *r*^het^ represents the heterogeneous reaction rate. Furthermore, *T*(*t*, *x*, ρ) and *T*_*S*_(*t*, *x*, ρ) are the absolute temperatures of the mobile phase and the solid phase, λ_*x*,*i*_ and λ_ρ,*i*_ represent the column’s axial and radial thermal conductivity coefficients respectively, Δ*H*_*A*,*j*_ is the *j*^th^ component heat of adsorption, *c*_*f*_ = ρ^*L*^c_*p*_^*L*^ and *c*_*e*_ = ρ^*S*^c_*p*_^*S*^, while ρ^*L*^ and ρ^*S*^ represent the density per unit volume in the mobile and solid phase, respectively. *c*_*p*_^*L*^ and *c*_*p*_^*S*^ are the corresponding heat capacities of the liquid and solid phases, *h*_*p*_ is the overall rate of heat transfer within mobile and solid phases, and Δ*H*_*R*_ is the heat of reaction. The stoichiometric coefficient ν_*i*_ is positive for products and negative for reactants. Based on the classical Van’t Hoff equation, an adsorption isotherm relates *q*_i_^*^ to liquid-phase concentrations and solid-phase temperature as follows:
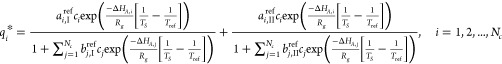
5where *a*_*i*,I_^ref^ and *a*_*i*,II_^ref^ symbolize, component-wise, Henry’s constants for the adsorption site I and site II, respectively, at the reference temperature *T*_ref_, *b*_*j*,I_^ref^ and *b*_*j*,II_^ref^ quantify the component-wise extent of nonlinearity for the adsorption site I and site II, respectively, and *R*_*g*_ represents the universal gas constant. The reaction rate in a typical heterogeneously catalyzed reactor considering the reversible reaction of the form *c*_3_ ⇄ *c*_1_ + *c*_2_ is expressed as
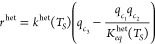
6In the above equation, *q*_*c*3_ is the concentration of reactant in the solid phase, and *q*_*c*1_, and *q*_*c*2_ symbolize the concentrations of products in solid phase, *K*_*eq*_^het^(T_*S*_) represents the chemical reaction equilibrium constant, and *k*^het^(T_*S*_) denote the forward heterogeneous reaction rate constant. The Arrhenius equation describes the effect of temperature on the chemical reaction rate as an exponential function of absolute temperature using activation energy *E*_Act_^het^. The expression of the Arrhenius equation is
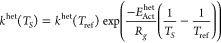
7where *E*_Act_^het^ is the activation energy. The chemical reaction equilibrium constant is expressed as
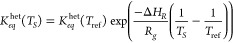
8

## Simplification of Mathematical Model

Dimensional analysis is a mathematical technique that helps determine a systematic arrangement of the variables in the physical relationship, combining dimensional variables to form nondimensional parameters. It is based on the principle of nonhomogeneity and is useful for presenting experimental results in concise form and reducing roundoff errors during computer simulations.^67^ The following new dimensionless variables are embedded into the model equations to minimize the number of involved variables:
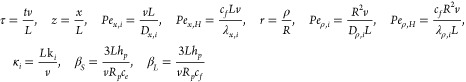
9The symbols *Pe*_*x*,*i*_ and *Pe*_*x*,*H*_ are the mass and heat transfer Peclet numbers along with the axial position of the cylindrical reactor, respectively, whereas the Peclet numbers for mass and heat transfer along with the radial position of the reactor are signified as *Pe*_ρ,*i*_ and *Pe*_ρ,*H*_, and *L* is the length of cylindrical reactor. By incorporating the above-defined scaled parameters, the system of governing equations, eqs 1–4, for 2D-RLKM in dimensionless form is given as follows:
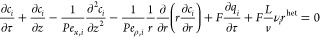
10
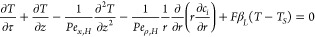
11
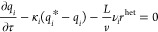
12
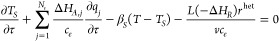
13The PDEs system presented in eqs 10–13 has to be equipped with appropriate initial and boundary conditions for the specified choice of closed form solution.

### Initial Conditions (ICs)

The mass and energy profiles are anticipated to satisfy the initial condition stated below:
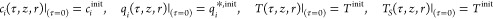
14where *c*_*i*_^init^ and *q*_i_^*,init^ represent the constant initial equilibrated concentrations in the mobile and solid phases, respectively, and *T*^init^ is the initial equilibrated temperature, which is set to be equal to the reference temperature for the current study *T*^init^ = *T*^ref^.

### Boundary Conditions (BCs)

The column structure is indispensable in analyzing the unexpected tailing behavior in a chromatographic process. The division of the column inlet could improve the column’s radial heterogeneity and efficiency in inner and outer annular regions. Various inflow conditions are assessed at the reactor inlet employing the Dankwert’s BCs. The inner circular region injections are described as follows:
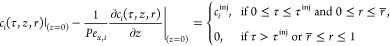
15
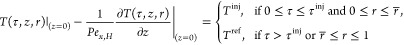
16whereas, the definition of the outer annular ring injections is as follows:
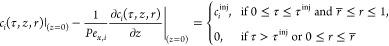
17
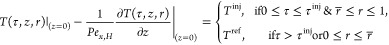
18Here, the symbol τ^inj^ is the dimensionless injection time, *c*_*i*_^inj^ is used to denote the injected concentration of the component *i*, and *T*^inj^ is the temperature of the injected component. Moreover, *r̅* = ρ̅/*R*, where ρ̅ stands for the inner zone radius. For injection across the entire cross sectional area of the reactor inlet, either set *r̅* = 0 in eqs 16 and 18 or set *r̅* = 1 in eqs 15 and 16. The BCs given in eqs 16 and 18 allow the change of injected sample temperature, which could be different from bulk phase temperature *T*^ref^. Neuman BCs at the right end of the reactor are defined as
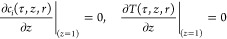
19The radial BCs taking into account the radial profile’s symmetry at *r* = 0 and the column’s wall impermeability at *r* = 1 are expressed as
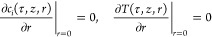
20
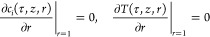
21

## Implementation of HR-FVM

Numerical simulations of the nonlinear chromatographic problem presented in this work are challenging due to the occurrence of steep concentration fronts.^47,59,68^ Consequently, the optimization and real-time control of chromatographic processes require highly reliable and effective numerical methods. Appendix I presents the detailed derivation of HR-FVM to approximate the nonisothermal 2D-RLKM.

## Validation of Numerical Results through Consistency Test

Suitable performance criteria are required to optimize the performance of the considered nonisothermal reactive chromatographic process. In practice, chromatographic reactors still lack experimental evidence for validating their mathematical models. Consequently, we performed the following integral-consistency tests to demonstrate the reliability of the numerical algorithm formulated for the current model equations. Since the law of conservation applies to each chemical reaction, therefore the total amount of injected concentration is conserved throughout the course of the reaction process. The conservation constraint is desired to measure the extent to which a reversible reaction of the form *c*_3_ ⇄ *c*_1_ + *c*_2_ proceeds. Let ξ denote the integrated extent of reaction, which indicates all changes in mole numbers caused by the chemical reaction:
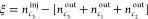
22where *n*_*ci*_ is the number of moles of *i*^*th*^ component of the mixture and *n*_*c*__*i*_^inj^ = *c*_*i*_^inj^*V*^inj^ represents the amount of *i*^*th*^ component in moles, inserted at the column’s inlet over an injection time τ^inj^, and *V*^inj^ denotes the injected volume of reactant. In the current analysis, we considered *c*_1_^inj^ = 0 = *c*_2_^inj^, thus *n*_*c*__2_^inj^ = 0 = *n*_*c*__3_^inj^. Moreover, the following integral formula can be utilized to compute the number of moles of reactants and products at the column outlet:
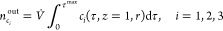
23In the above formula, τ^max^ is the final simulation time, *V̇* denotes the volumetric flow rate associated with the linear velocity *v*. The change in number of moles of the *i*^*th*^ component of the reaction is expressed as
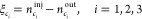
24Using the three values of ξ_*ci*_ derived from eq 24, the standard deviation can be computed as follows:
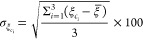
25where, ξ̅ represents the mean value of ξ_*c*1_, ξ_*c*2_, and ξ_*c*3_. If the mass balance equations respect reaction stoichiometry, this standard deviation should approach zero. Since the energy balance for a nonisothermal chemical reactor determines the reactor’s temperature, an energy-based evaluation of the underlying chemical and segregation processes can be accomplished by the comparison of enthalpies leaving and entering the system, which are represented symbolically by Δ*H*^out^ and Δ*H*^inj^, respectively, and are given as
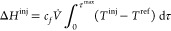
26
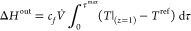
27For the current analysis, *T*^ref^ = *T*^inj^, hence Δ*H*^out^ = 0. Furthermore, for a sufficiently large τ^max^, in the case of a complete cycle of adsorption–desorption process, the overall sorption effects will be nullified.
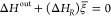
28Hence the fulfillment of eq 28 exploits the accuracy of the obtained numerical results. However, the right hand side of eq 28 might not be precisely zero due to multiple sources of errors in the course of applying any numerical scheme. Let Δ*H*^error^ represent the error in numerical simulation, then the precision of our proposed numerical method can be determined by the expression
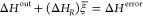
29The integral of mass and energy balances converges for a small value of the Δ*H*^error^. The relative error percentage in energy calculation is represented as
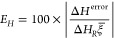
30Moreover, the percentage rate of reactant conversion can be calculated using the following formula:
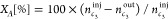
31

## Results and Discussion on Numerical Case Studies

Several numerical test studies are performed in this section to determine the effect of various physical parameters on the separation, conversion, and temperature fluctuation under Bi-Langmuir adsorption conditions in 2D nonisothermal liquid reactive chromatography. To emphasize the impact of injection mode in the considered test problems, the reactor radius is set to R = 1.25 cm and the outer annular ring is set to ρ = 0.8838 cm. Some assumptions have been made in conducting numerical tests to facilitate the simulation process. The dispersion coefficients of the space variables *D*_*x*,*i*_, *D*_ρ,*i*_ and rate constant *k*_*i*_ of mass transfer are deemed to have the same value for all the sample components. The reactant is fed through the inner cylindrical core of the column. Furthermore, unless otherwise specified, the value of enthalpy of adsorption is also assumed to be the same for all the components of the sample that is Δ*H*_*A*,*i*_ = Δ*H*_*A*_ for *i* = 1, 2, 3. The list of different parameters involved in this study is provided in Table 1. The estimated standard physical parameters used in the simulation study are based on the theoretical research conducted by Tien.^42^ However, it should be noted that experimental results for 2D-nonisothermal liquid reactive chromatography are currently unavailable.

**Table 1:** List of Model Parameters Used in the Simulation Studies

Parameters	Values
Column length	*L* = 25.0 cm
Column porosity	ϵ_*c*_ = 0.25
Column radius	*R* = 1.25 cm
Dispersion coefficients	*D*_*x*,*i*_ = 0.00001,*D*_ρ,*i*_ = 0.1 cm^2^/min(*i* = 1, 2, 3)
Injection time	*t*^inj^ = 10.0 min
Maximum simulation time	*t*^max^ = 100 min
Reference temperature	*T*^ref^ = 300 K
Initial concentrations	*c*_*i*_^init^ = 0 mol/L
Inlet concentration of component 1	*c*_1_^inj^ = 0.0 mol/L
Inlet concentration of component 2	*c*_2_^inj^ = 0.0 mol/L
Inlet concentration of component 3	*c*_3_^inj^ = 0.5 mol/L
Henry constants for component 1	*a*_1,I_^ref^ = 5.0, *a*_1,II_^ref^ = 6.0
Henry constants for component 2	*a*_2,I_^ref^ = 1.0, *a*_2,II_^ref^ = 1.0
Henry constants for component 3	*a*_3,I_^ref^ = 3.0, *a*_3,II_^ref^ = 4.0
Reference values of adsorption energy coefficients	*b*_*i*,I_^ref^ = 1.0, *b*_*i*,II_^ref^ = 2.0
Specific heat for solid phase	*c*_*e*_ = 4 J/Kcm^3^
Specific heat for mobile phase	*c*_*f*_ = 4 J/Kcm^3^
Radius of the particle	*R*_*p*_ = 0.2 cm
Axial heatconductivity coefficient	λ_*x*,*i*_ = 4*D*_*x*,*i*_ = 0.00004 kJ cm^–1^ min^–1^
Radial heat conductivity coefficient	λ_ρ,*i*_ = 4*D*_ρ,*i*_ = 0.4 kJ cm^–1^ min^–1^
Mass transfer coefficient	k_*i*_ = 100 min^–1^
Heat transfer coefficient	*h*_*p*_ = 24 W/cm^2^ K
Velocity	*v* = 100cm/min
Activation energy	*E*_Act_ = 60.0 kJ/mol
Heterogeneous reaction rate constant	*k*^het^ = 5 min^–1^
Chemical reaction equilibrium constant	*k*^het^_eq_ = 0.8333 mol/L

### Isothermal Conditions (Δ*H*_*A*,*i*_ = 0, Δ*H*_*R*_ = 0 kJ/mol)

**Figure 2:**
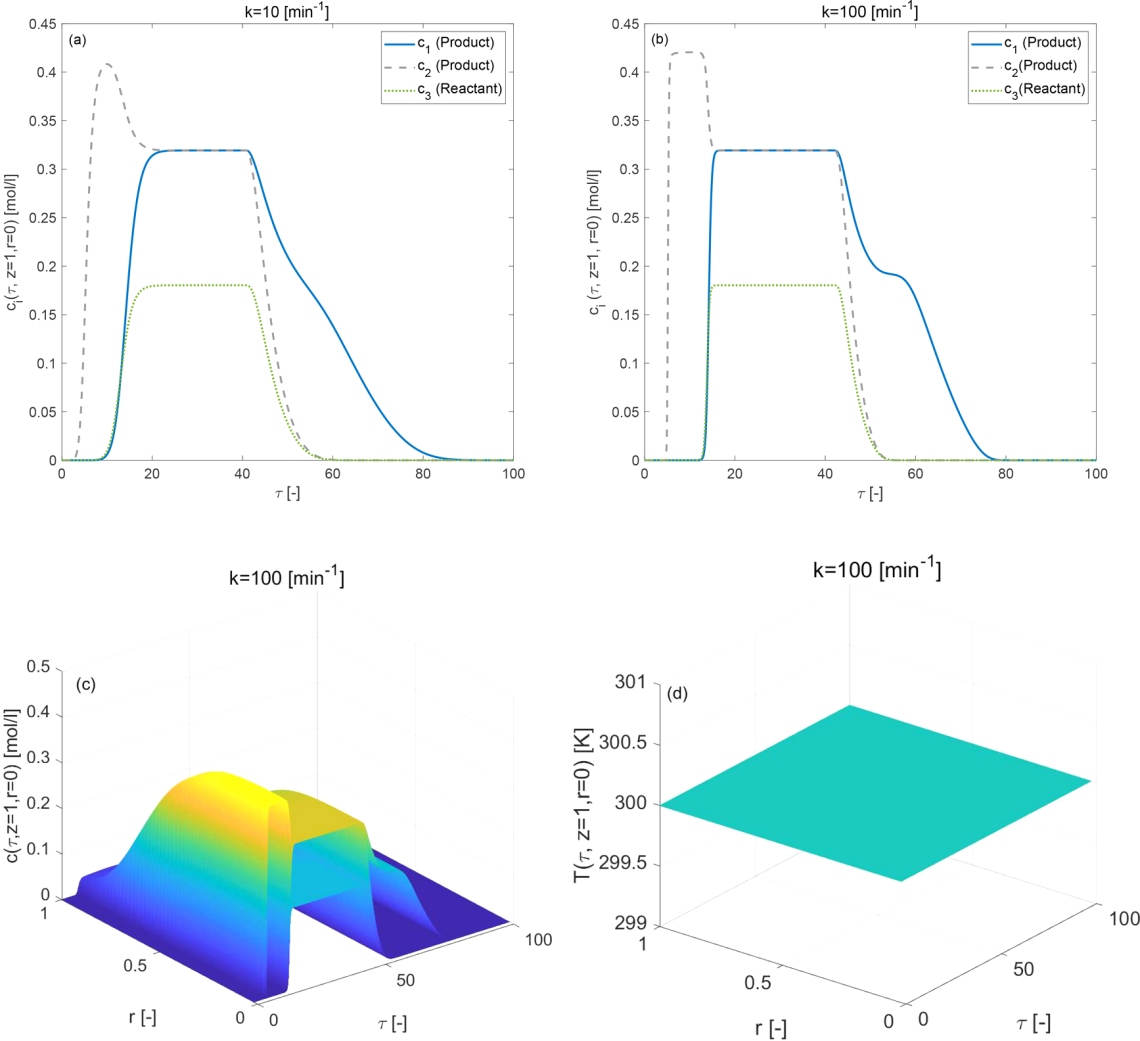
Reference isothermal case for three-component reaction considering Bi-Langmuir adsorption isotherm. (a,b) Concentration profiles are generated for two different values of mass transfer coefficient *k*. (c,d): 3D plots of concentration and temperature profiles for *k* = 100 [min^–1^]. Here Δ*H*_*R*_ = 0 = Δ*H*_*A*,*i*_ kJ/mol, *b*^ref^_i,I_ = 1, *b*^ref^_i,II_ = 2 for *i* = 1, 2, 3.

**Figure 3:**
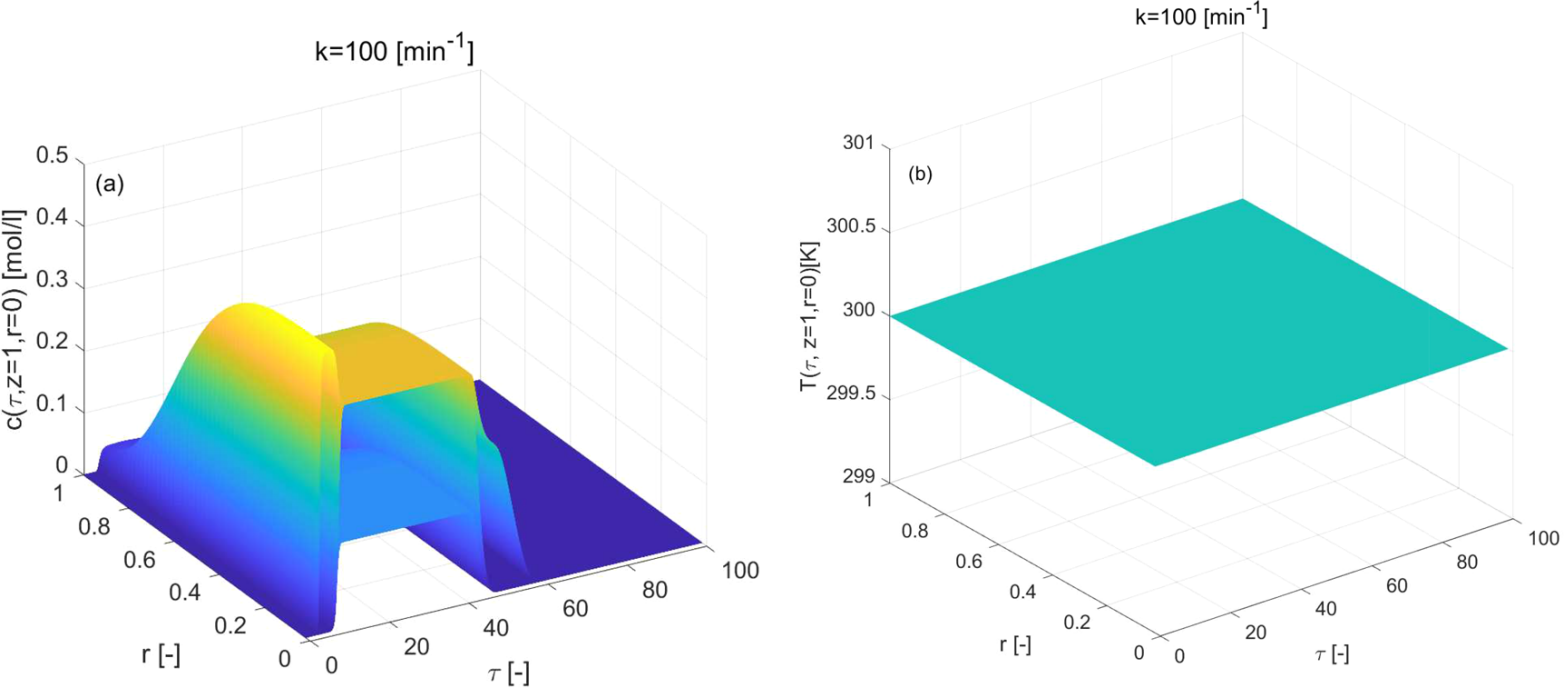
Reference isothermal case for three-component reaction considering Langmuir adsorption isotherm. (a,b) 3D plots of concentration and temperate profiles are generated for *k* = 100 min^–1^. Here Δ*H*_*R*_ = 0 = Δ*H*_*A*,*i*_ kJ/mol, *a*^ref^_i,II_ = 0, *b*^ref^_i,I_ = 1, *b*^ref^_i,II_ = 0 for *i* = 1, 2, 3.

In this first case study, the effects of Bi-Langmuir adsorption isotherm for two incompatible values of the mass transfer rate, i.e., *k* = 10 min^–1^, and *k* = 100 min^–1^ and the effects of the Langmuir (i.e *a*_*i*,II_^ref^ = 0 and *b*_*i*,II_^ref^ = 0 in eq 5) adsorption isotherm for *k* = 100 min^–1^ are investigated under isothermal conditions (Δ*H*_*A*,*i*_ = 0, Δ*H*_*R*_ = 0 kJ/mol). The present case study will serve as a benchmark to inspect other simulation results. Figure 2(a–d) demonstrates the impact of the Bi-Langmuir adsorption isotherm and Figure 3(a,b) displays the impact of the Langmuir adsorption isotherm, respectively, on the eluted profiles exhibiting the distribution of concentration for reactant and products, and temperature, for the considered reversible reaction of the type *c*_3_ ⇄ *c*_1_ + *c*_2_. The Langmuir isotherm assumes uniform solute absorption through the monolayer surface of the stationary phase. On the other hand, the double-adsorption sites on the surface of the stationary phase are not uniform, and consequently, the solutes exhibited varying behavior in the column, which affected peak tailing. The purity and yield in preparative chromatography are influenced by the peak tailings. The reaction has occurred in each case, and the products are produced due to the solid bed’s catalytic nature. However, inadequate separation and conversion are visible for the selected values of adsorption equilibrium constants. As the magnitude of *k* increases, the presence of stretched tail, sharp fronts, and typical Bi-Langmuir behavior in the elution profiles is visible from the plots of Figure 2. The value of *k* has an effect comparable to diffusion on the eluted profiles. It has been observed that the process is still not in equilibrium for small values of *k*, with the convection process being dominated by the diffusion phenomena. As expected for the current case, no variations in temperature profiles have been identified, and the mass transfer coefficient has no effect on the average retention time. It is worth mentioning here that the conversion rate of the reactant is 78[%] in the case of Langmuir isotherm, which reduces further to 75[%] (see Table 2) in the case of Bi-Langmuir isotherm as a result of the difference in the adsorption activity.

**Table 2:** Validation of Numerical Results through Integral Consistency Test for Distinct Values of Δ*H*_*R*_ and Δ*H*_*A*,*i*_ = 0 kJ/mol, *k* = 100 min^–1^, *E*_Act_ = 60 kJ/mol

	ξ_*c*1_	ξ_*c*2_	ξ_*c*3_	σ_ξ,*i*_	χ_*c*3_	Δ*H*^out^	Δ*H*^error^	*E*_*H*_
Parameters	(mol)	(mol)	(mol)	(%)	(%)	(kJ)	(kJ)	(%)
Δ*H*_*R*_ = 0	0.0075	0.0075	0.0075	0.0017	75	0	0	
Δ*H*_*R*_ = −40	0.0073	0.0073	0.0073	0.0016	73	0.0542	–0.2385	0.8148
Δ*H*_*R*_ = −80	0.0068	0.0068	0.0068	0.0016	68	0.0980	–0.4481	0.8206
Δ*H*_*R*_ = 40	0.0073	0.0073	0.0073	0.0017	73	–0.0541	0.2378	0.8147

### Effects of Nonzero Enthalpy of Reaction (Δ*H*_*A*,*i*_ = 0 kJ/mol, Δ*H*_*R*_ ≠ 0 kJ/mol)

**Figure 4:**
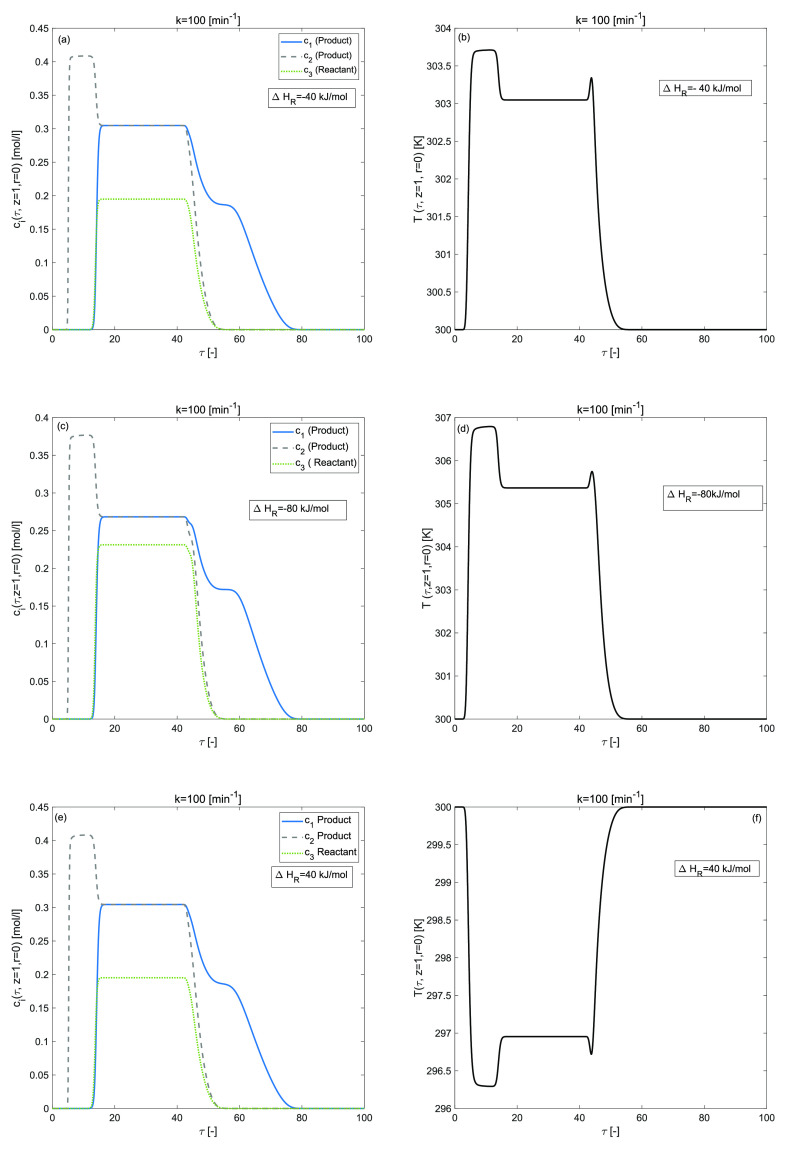
Effects of enthalpy of reaction on concentration and temperature profiles during the three component reaction. (a,b) Δ*H*_*R*_ = −40 kJ/mol, (c,d) Δ*H*_*R*_ = −80 kJ/mol, (e,f) Δ*H*_*R*_ = 40 kJ/mol, and Δ*H*_*A*,*i*_ = 0 kJ/mol, *E*_*Act*_ = 60 kJ/mol, *b*^ref^_i,I_ = 1, *b*^ref^_i,II_ = 2 for *i* = 1, 2, 3.

**Figure 5:**
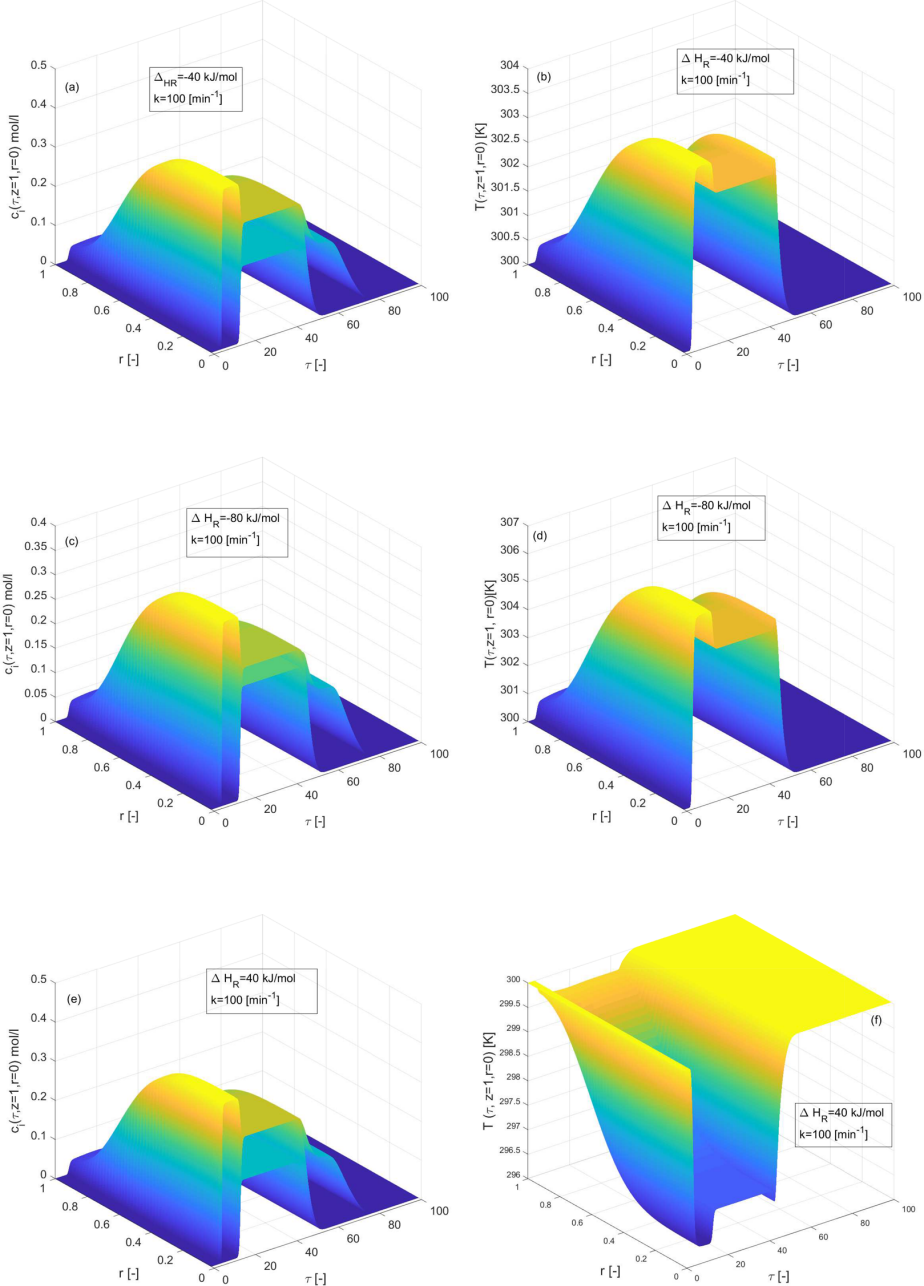
3D plots depicting the effects of enthalpy of reaction on concentration and temperature profiles during the three component reaction. (a,b) Δ*H*_*R*_ = −40 kJ/mol, (c,d) Δ*H*_*R*_ = −80 kJ/mol, (e,f) Δ*H*_*R*_ = 40 kJ/mol, and Δ*H*_*A*,*i*_ = 0 kJ/mol, *E*_*Act*_ = 60 kJ/mol, *b*^ref^_i,I_ = 1, *b*^ref^_i,II_ = 2 for *i* = 1, 2, 3.

In this numerical test, the influence of nonzero enthalpy of reaction Δ*H*_*R*_ on concentration and temperature profile is investigated. The 2D and 3D plots are presented in Figures 4 and 5 for an exothermic-reaction, Δ*H*_*R*_ = −40 kJ/mol, Δ*H*_*R*_ = −80 kJ/mol and for an endothermic-reaction for Δ*H*_*R*_ = 40 kJ/mol, respectively, while the enthalpy of adsorption Δ*H*_*A*,*i*_ = 0 kJ/mol. Temperature fluctuations in the temperature profile and an asymmetrically shaped tailing elution concentration profile with a considered overloaded sample were revealed in 2D and 3D plots. For Δ*H*_*R*_ = −40 kJ/mol and Δ*H*_*R*_ = 40 kJ/mol, the conversion rate of reactant is 73[%] which decreases to 68[%] for Δ*H*_*R*_ = −80 kJ/mol as given in Table. 2. The conversion rate decreases due to the nonlinearities in the reaction term and in the adsorption isotherm. The concentration of products increases for a smaller value of enthalpy of reaction, as depicted in Figures 4(a) and 5(a). It can be noticed that the temperature has increased to its maximum value as the magnitude of Δ*H*_*R*_ has increased. This phenomena is depicted in Figures 4(b and d) and 5(b and d). Furthermore, the nonlinearity effects are significant for all values of reaction enthalpy. The 3D plots depicts eluent behavior at the column’s radial center.

### Effects of Nonzero Enthalpy of Adsorption (Δ*H*_*A*,*i*_ ≠ 0 kJ/mol, Δ*H*_*R*_ = 0 kJ/mol)

**Figure 6:**
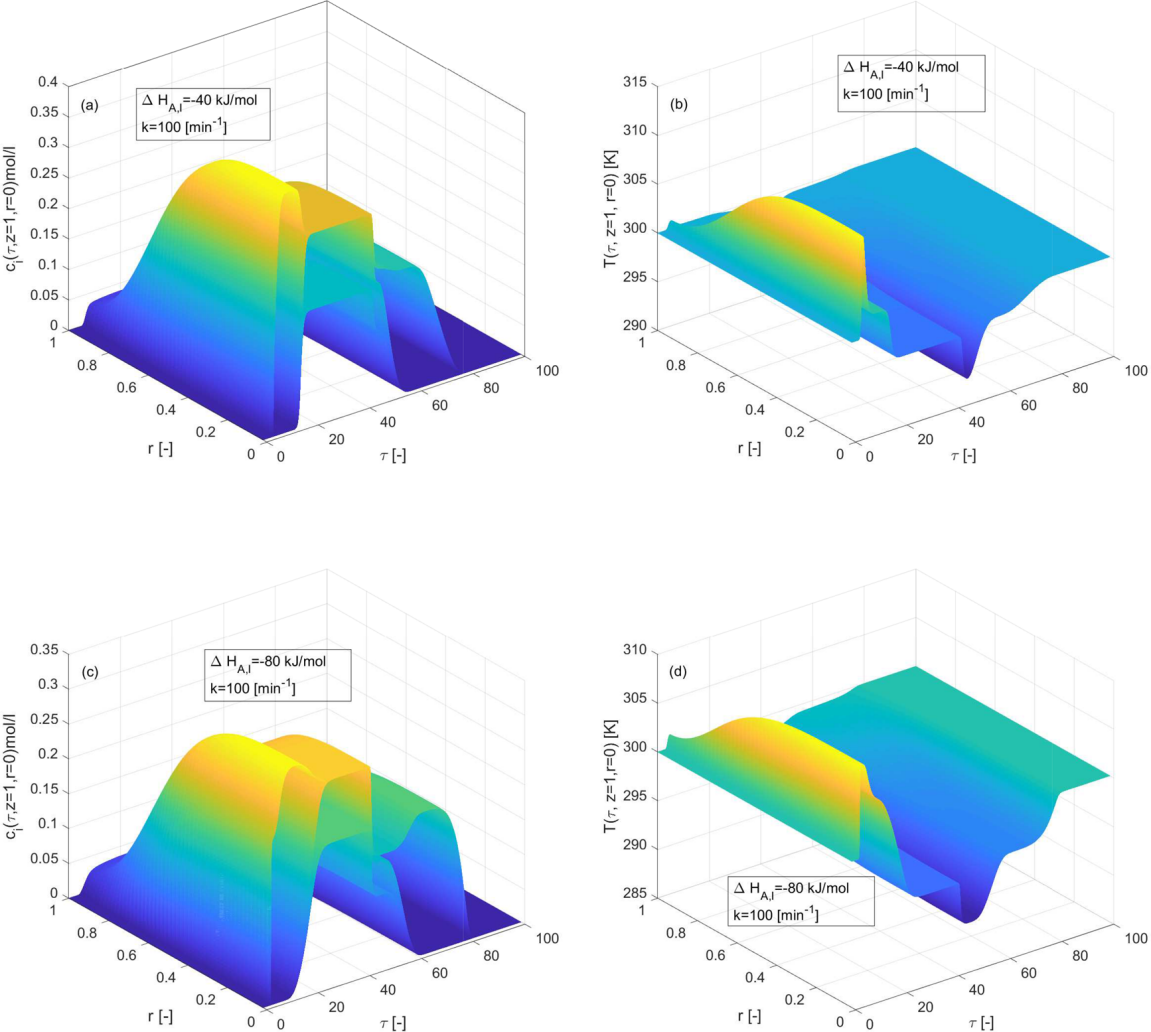
3D-plots depicting the effects of enthalpy of adsorption on concentration and temperature profiles during the three component reaction. (a,b) Δ*H*_*A*,*i*_ = −40 kJ/mol, (c,d) Δ*H*_*A*,*i*_ = −80 kJ/mol, and Δ*H*_*R*_ = 0 kJ/mol, *E*_*Act*_ = 60 kJ/mol, *b*^ref^_i,I_ = 1, *b*^ref^_i,II_ = 2 for *i* = 1, 2, 3.

The effect of nonzero Δ*H*_*A*,*i*_ while Δ*H*_*R*_ = 0 kJ/mol is quantified in Figure 6. The enthalpy of adsorption is taken to be the same for each component in the current test problem. The enthalpy of adsorption provides thermodynamic insight into the reaction-separation process since product separation from reactant based on adsorption relies on the equilibrium loading differences of the mixture components, which is highly dependent on temperature fluctuations, which mostly result from the change in enthalpy of adsorption. The quantitative evidence presented in Table 3 demonstrates that increasing the magnitude of the enthalpy of adsorption results in relatively less reactant conversion and product separation. The rate of conversion is 74[%], when Δ*H*_*A*,*i*_ is −40 kJ/mol and scales down to 73[%] when Δ*H*_*A*,*i*_ is −80 kJ/mol. The components residence time has increased for Δ*H*_*A*,*i*_ = −80 kJ/mol, and one of the products has begun eluting faster. Moreover, increasing the value of enthalpy of adsorption led to the observation of self-sharpening, asymmetric concentration and temperature profiles, evincing a heterogeneity in the adsorption function of the reactant and the products for two independent adsorption sites.

**Table 3:** Validation of Numerical Results through Integral Consistency Test for Distinct Values of Δ*H*_*A*,*i*_ and Δ*H*_*R*_ = 0 kJ/mol, *k* = 100 min^–1^, *E*_Act_ = 60 kJ/mol

	ξ_*c*1_	ξ_*c*2_	ξ_*c*3_	σ_ξ,*i*_	χ_*c*3_	Δ*H*^out^	Δ*H*^error^	*E*_*H*_
Parameters	(mol)	(mol)	(mol)	(%)	(%)	(kJ)	(kJ)	(%)
Δ*H*_*A*_ = 0	0.0075	0.0075	0.0075	0.0017	75	0	0	-
Δ*H*_*A*_ = −40	0.0074	0.0074	0.0074	0.0017	74	–0.0557	–0.0557	-
Δ*H*_*A*_ = −80	0.0073	0.0073	0.0073	0.0017	73	–0.1090	–0.1090	-

### Synergetic Effects of Nonzero Reaction and Adsorption Enthalpies (Δ*H*_*R*_ ≠ 0 kJ/mol), Δ*H*_*A*,*i*_ ≠ 0 kJ/mol

**Figure 7:**
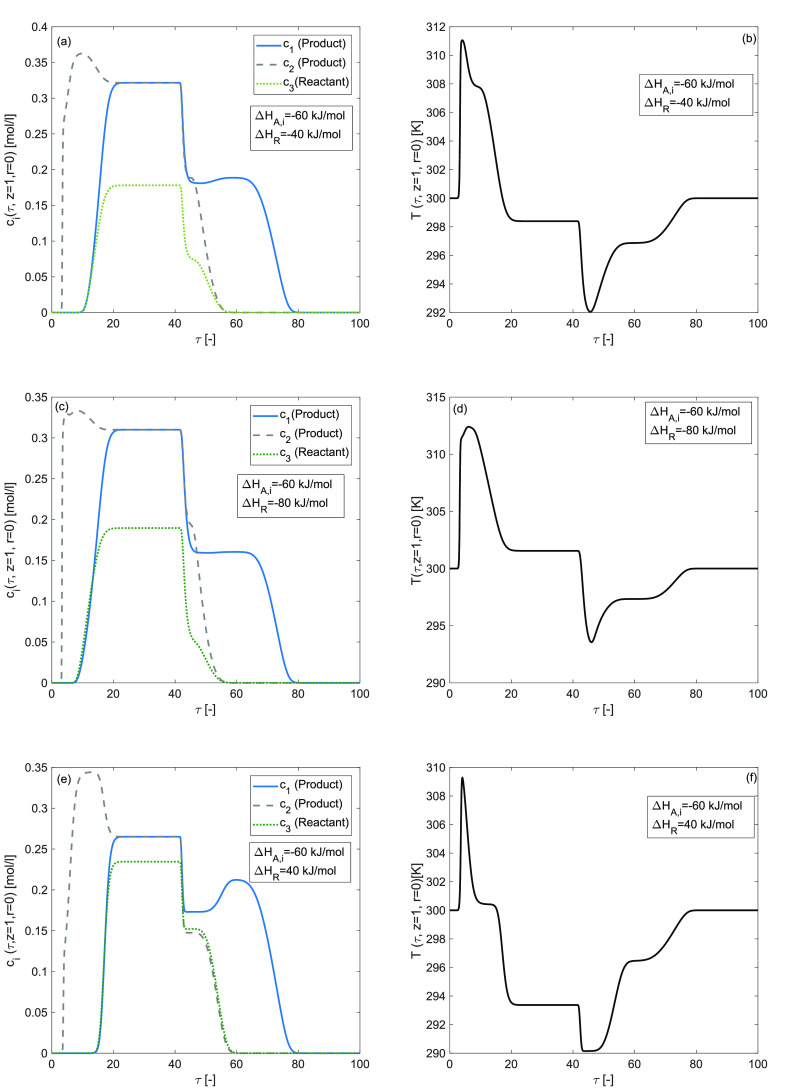
Effect of both enthalpies of reaction and adsorption: Δ*H*_*A*,*i*_ = −60 kJ/mol, for *i* = 1, 2, 3. (a,b) Δ*H*_*R*_ = −40 kJ/mol, (c,d) Δ*H*_*R*_ = −80 kJ/mol, (e,f) Δ*H*_*R*_ = 40 kJ/mol, *E*_*Act*_ = 60 kJ/mol, *b*^ref^_i,I_ = 1, *b*^ref^_i,II_ = 2.

**Figure 8:**
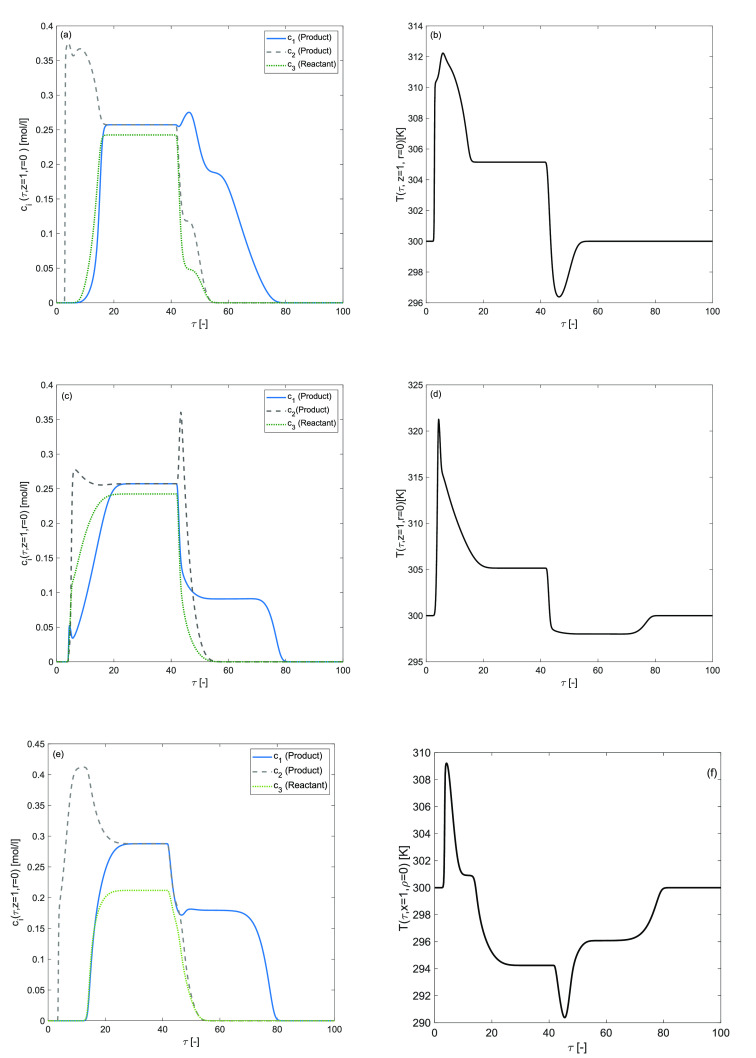
Effects of component wise enthalpy of adsorption on concentration and temperature profiles, Δ*H*_*R*_ = −80 kJ/mol. (a,b) Δ*H*_*A*,1_ = 0, Δ*H*_*A*,2_ = Δ*H*_*A*,3_ = −80 kJ/mol, (c,d) Δ*H*_*A*,1_ = −80 = Δ*H*_*A*,3_, Δ*H*_*A*,2_ = 0 kJ/mol, (e,f) Δ*H*_*A*,1_ = −80 = Δ*H*_*A*,2_, Δ*H*_*A*,3_ = 0 kJ/mol, *E*_Act_ = 60 kJ/mol, *k* = 100 min^–1^, *b*^ref^_i,I_ = 1, *b*^ref^_i,II_ = 2 for *i* = 1, 2, 3.

**Figure 9:**
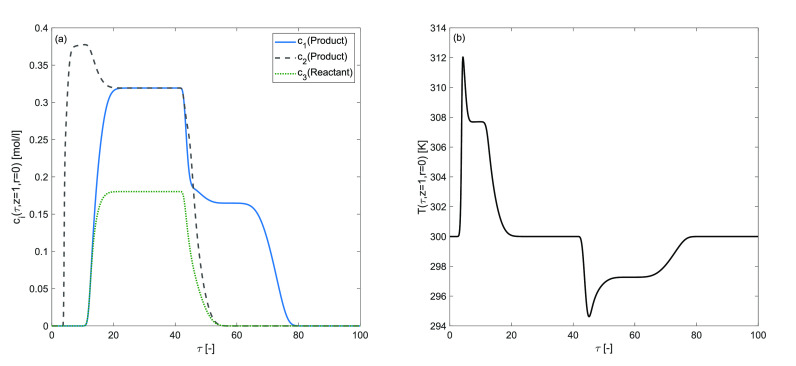
Effects of distinct enthalpy of adsorption on concentration and temperature profiles. (a,b) Δ*H*_*R*_ = −80 kJ/mol, Δ*H*_*A*,1_ = −60 kJ/mol, Δ*H*_*A*,2_ = −40 kJ/mol, Δ*H*_*A*,3_ = −20 kJ/mol, k = 100 min^–1^.

Synergetic effects of reaction and adsorption enthalpies are considered in this numerical test problem to quantitatively evaluate the efficiency of a nonisothermal chromatographic reactor in Bi-Langmuir adsorption conditions. The simulation results are provided in Figures 7, 8, and 9. Figure 7(a–f) evaluates the effects of fixed enthalpy of adsorption Δ*H*_*A*,*i*_ = −60 kJ/mol for *i* = 1, 2, 3 along with variable enthalply of reaction. Figure 7(a,b) reflect an evident decrease in the reactant *c*_3_ peak height and a significant increase in the products *c*_1_, *c*_2_ peak heights. As a result, the forward reaction is enhanced, and more reactant is further converted. The percentage conversion of reactant is 76[%] in the case when Δ*H*_*A*,*i*_ = −60 kJ/mol and Δ*H*_*R*_ = −40 kJ/mol. The quantitative figures listed in Table 4 indicate the accuracy of the obtained numerical solutions. The computational results of the current numerical test revealed that the conversion and temperature have increased with a smaller value of enthalpy of reaction. The effects of fixed Δ*H*_*R*_ = −80 kJ/mol with a variable Δ*H*_*A*,*i*_ in the range [−80,0] are shown in Figure 8(a–f). In Figure 8(a,b) the enthalpy of adsorption Δ*H*_*A*,1_ for the first product, in Figure 8(c,d) the enthalpy of adsorption Δ*H*_*A*,2_ for the second product, and in Figure 8(e,f) the enthalpy of adsorption Δ*H*_*A*,3_ for the reactant is assigned as zero, respectively. Now, component-wise, indifferent enthalpy of adsorption has a prominent impact on the elution profiles. The extent of adsorption enthalpy impacts the component’s affinity. Therefore, the components inside the reactor speed up when the adsorption enthalpy is high. Table 5 quantitatively evaluates the effects of indifferent enthalpy of adsorption and reaction. The reactant percentage conversion is high, reaching up to 72[%] when Δ*H*_*A*,1_ = −80 = Δ*H*_*A*,2_, Δ*H*_*A*,3_ = 0 kJ/mol and Δ*H*_*R*_ = −80 kJ/mol (see Figure 8(e,f)). In each case, significant variations appear in the temperature profile due to the considered adsorption and desorption processes. In Figure 8(c,d), the height of the desorption front slows and spreads, and the adsorption front of the temperature profile becomes narrower and elutes earlier. The mean residence time for concentration and temperature profiles is the same in each case.

**Table 4:** Performance Evaluation for the Synergetic Effects of Enthalpies of Adsorption and Reaction, Δ*H*_*A*,*i*_ = −60 kJ/mol, *k* = 100 min^–1^, *E*_Act_ = 60 kJ/mol

	ξ_*c*1_	ξ_*c*2_	ξ_*c*3_	σ_ξ,*i*_	χ_*c*3_	Δ*H*^out^	Δ*H*^error^	*E*_*H*_
Parameters	(mol)	(mol)	(mol)	(%)	(%)	(kJ)	(kJ)	(%)
Δ*H*_*R*_ = −40	0.0076	0.0076	0.0076	0.0017	76	–0.0287	–0.3318	1.0946
Δ*H*_*R*_ = −80	0.0074	0.0074	0.0074	0.0017	74	0.0273	–0.5636	0.9538
Δ*H*_*R*_ = 40	0.0069	0.0069	0.0068	0.0017	68	–0.1236	0.1506	0.5492

**Table 5:** Performance Evaluation on the Basis of Indifferent Enthalpies of Adsorption with Δ*H*_*R*_ = −80 kJ/mol, *E*_*Act*_ = 60 kJ/mol, *k* = 100 min^–1^

	ξ_*c*1_	ξ_*c*2_	ξ_*c*3_	σ_ξ,*i*_	χ_*c*3_	Δ*H*^out^	Δ*H*^error^	*E*_*H*_
Parameters	(mol)	(mol)	(mol)	(%)	(%)	(kJ)	(kJ)	(%)
Δ*H*_*A*,1_ = 0	0.0069	0.0069	0.0068	0.0017	68	0.0980	–0.4506	0.8213
Δ*H*_*A*,2_ = 0	0.0064	0.0064	0.0063	0.0017	63	0.0887	–0.4210	0.8260
Δ*H*_*A*,3_ = 0	0.0073	0.0073	0.0072	0.0017	72	–0.1071	–0.6874	1.1846

The effects of distinct enthalpy of adsorption for each component are evaluated in Figure 9. The simulation results are produced by assuming Δ*H*_*A*,1_ = −60, Δ*H*_*A*,2_ = −40, Δ*H*_*A*,3_ = −20 kJ/mol, and Δ*H*_*R*_ = −80 kJ/mol. The increased rate of reaction and the conversion rate describe the prophesied rise in the profile of temperature. The percentage conversion of the reactant is 75[%]. The computational results of these numerical tests revealed that a comparatively smaller value of enthalpy of adsorption yields a better rate of conversion under Bi-Langmuir adsorption conditions.

### Effects of Different Adsorption Energies

**Figure 10:**
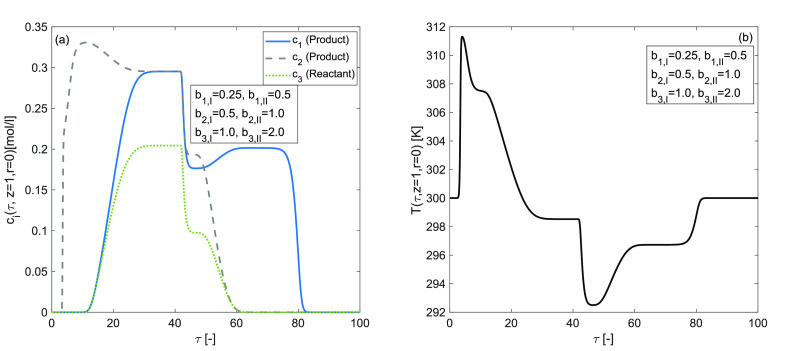
Effects of distinct adsorption energy coefficient for site I and for site II on concentration and temperature profiles. (a,b) Δ*H*_*R*_ = −40 kJ/mol, Δ*H*_*A*,*i*_ = −60 kJ/mol, *k* = 100 min^–1^.

The effects of the extent of the adsorption energy on the concentration profiles of each component are studied in Figure 10 to explore the complexity of entire reactive-adsorption process, which is determined by double-adsorption sites on the surface of the stationary phase and the intraparticle diffusion. The simulation results are produced by assuming *b*_1,I_^ref^ = 0.25, *b*_1,II_^ref^ = 0.5, *b*_2,I_^ref^ = 0.5, *b*_2,II_^ref^ = 1.0, *b*_3,I_^ref^ = 1.0, *b*_3,II_^ref^ = 2.0, Δ*H*_*A*,*i*_ = −60,kJ/mol, and Δ*H*_*R*_ = −40 kJ/mol. The predicted concentration plots in Figure 10 depict a bimodel shape, and the temperature profile exhibits a substantial increase in the peak height. The results of consistency analysis revealed that, assuming different adsorption energy coefficients for each component, the difference in adsorption activity between two independent adsorption sites for the eluent was enhanced. Thus, a larger quantity of the reactant is converted into products. In percentage terms, the reactant conversion rate is 74[%].

### Effects of Ratio *c*_*e*_/*c*_*f*_

**Figure 11:**
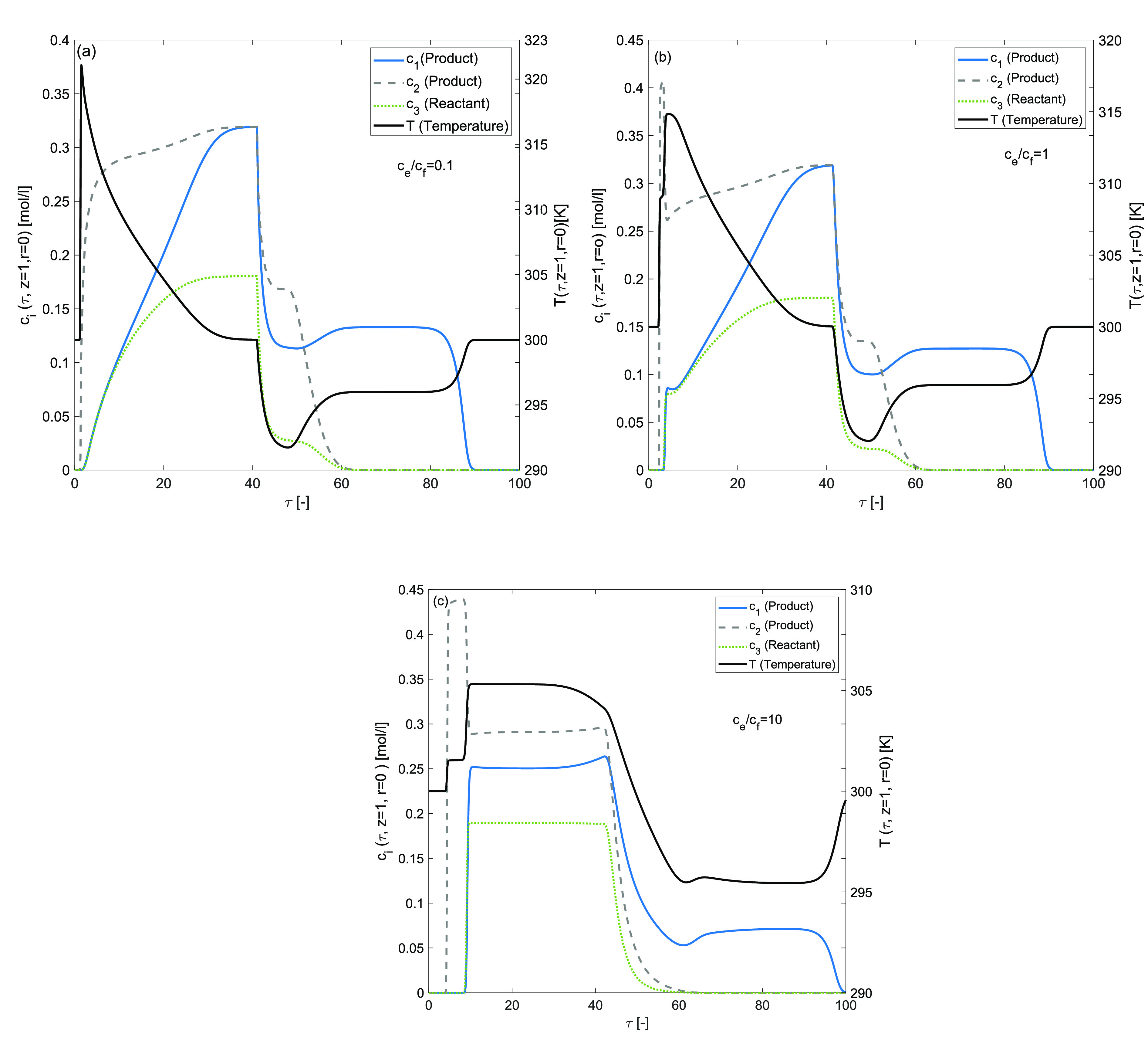
Effects of the ratio *c*_*e*_/*c*_*f*_ on concentration and temperature profiles during the three component reaction. (a) *c*_*e*_/*c*_*f*_ = 0.1, (b) *c*_*e*_/*c*_*f*_ = 1, (c) *c*_*e*_/*c*_*f*_ = 10, Δ*H*_*R*_ = −120 kJ/mol, Δ*H*_*A*,*i*_ = −120 kJ/mol, *E*_*Act*_ = 60 kJ/mol,*k*_*i*_ = 100 min^–1^, *b*^ref^_i,I_ = 1, *b*^ref^_i,II_ = 2 for *i* = 1, 2, 3.

The impact of changing density time heat capacity ratio  are studied in this numerical case study under the influence of larger magnitudes for both adsorption and reaction enthalpies. For the current numerical test problems, we have considered Δ*H*_*A*,*i*_ = −120 kJ/mol, Δ*H*_*R*_ = −120 kJ/mol, and *E*_Act_ = 60 kJ/mol. The simulation results are displayed in Figure 11. This ratio plays an important role in delineating the retention time and the propagation speed of concentration and temperature fronts inside the reactor. Figure 11(a), when , depicts a very steep, asymmetric, largest in magnitude, positive peak related to the temperature wave adsorption, while the negative peak related to desorption is diffusive. Moreover, the temperature wave is propagating at the fastest speed in comparison to the three concentration fronts. The average retention time for the concentration and the temperature front is the same in Figure 11(b), . The predicted profiles are coupled and are propagating at the same speed. Further, in this case, a larger quantity of reactant is converted into product. Figure 11(c), when , the temperature profile is diffusive and the adsorption peak of the temperature front is coupled with the concentration fronts. Comparing the coupling case to the decoupling case, temperature fluctuations are greater in the coupling case.

### Effects of Radial Dispersion Coefficients

**Figure 12:**
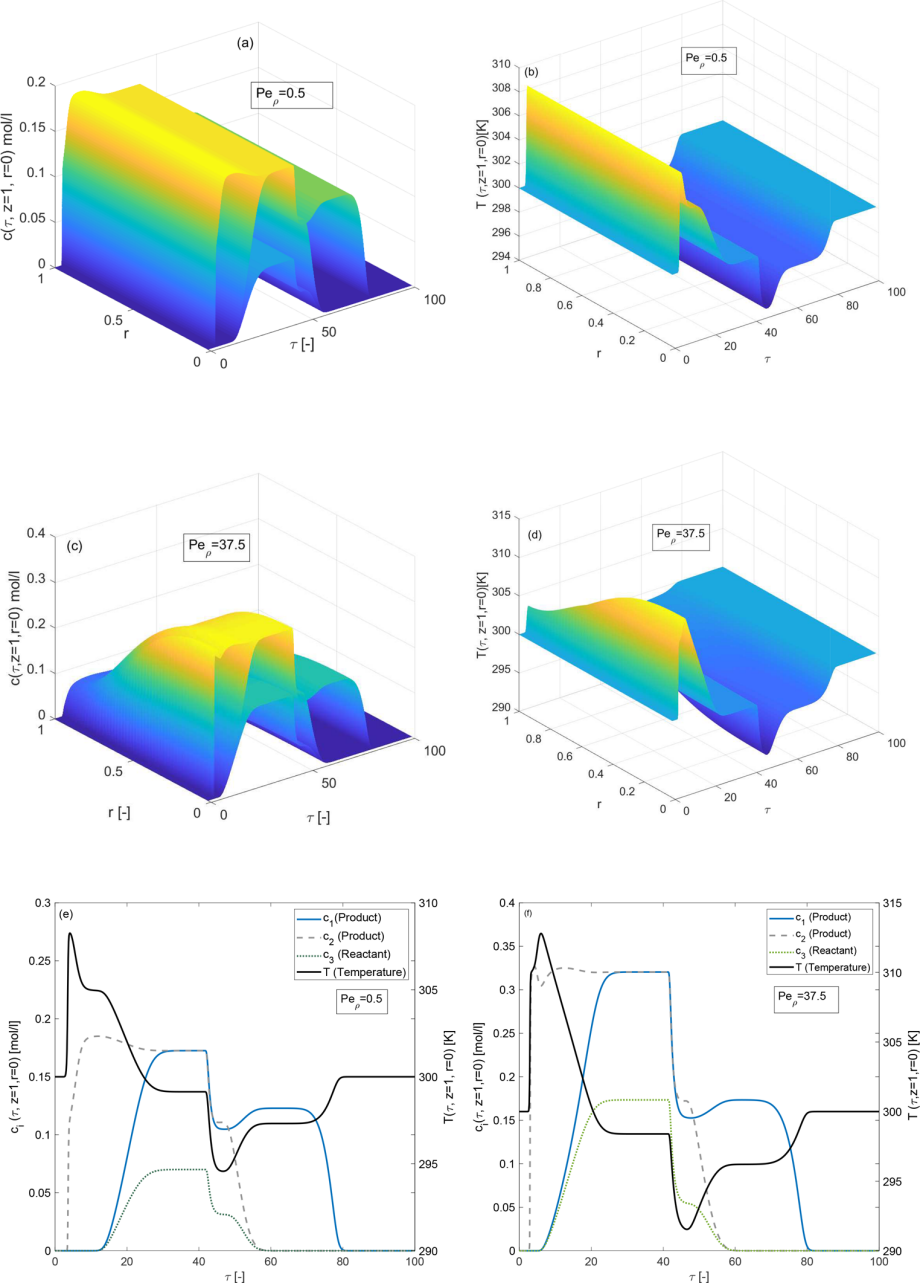
Effects of radial mass dispersion coefficient on concentration and temperature profiles, (a,b) *Pe*_ρ_ = 0.5, (c,d) *Pe*_ρ_ = 37.5, (e,f) *Pe*_ρ_ = 0.5, and Δ*H*_*R*_ = −60 kJ/mol, Δ*H*_*A*,*i*_ = −80 kJ/mol, *E*_Act_ = 60 kJ/mol, *k* = 100 min^–1^, *b*^ref^_i,I_ = 1, *b*^ref^_i,II_ = 2 for *i* = 1, 2, 3.

The impacts of radial mass dispersion coefficient *D*_ρ,*i*_ which is embedded in the dimensionless number  is discussed in Figure 12. The simulation results are generated for Δ*H*_*A*,*i*_ = −80 kJ/mol and Δ*H*_*R*_ = −60 kJ/mol for *Pe*_ρ,*i*_ = 0.5, the elution profile of each component remains constant across the radial position. The radial influences have been observed from the 3D plots for moderately higher value, i.e., for *Pe*_ρ,*i*_ = 37.5. In addition, the peak height of the temperature profile is high when *Pe*_ρ,*i*_ = 37.5, whereas the conversion rate is high when *Pe*_ρ,*i*_ = 0.5.

**Figure 13:**
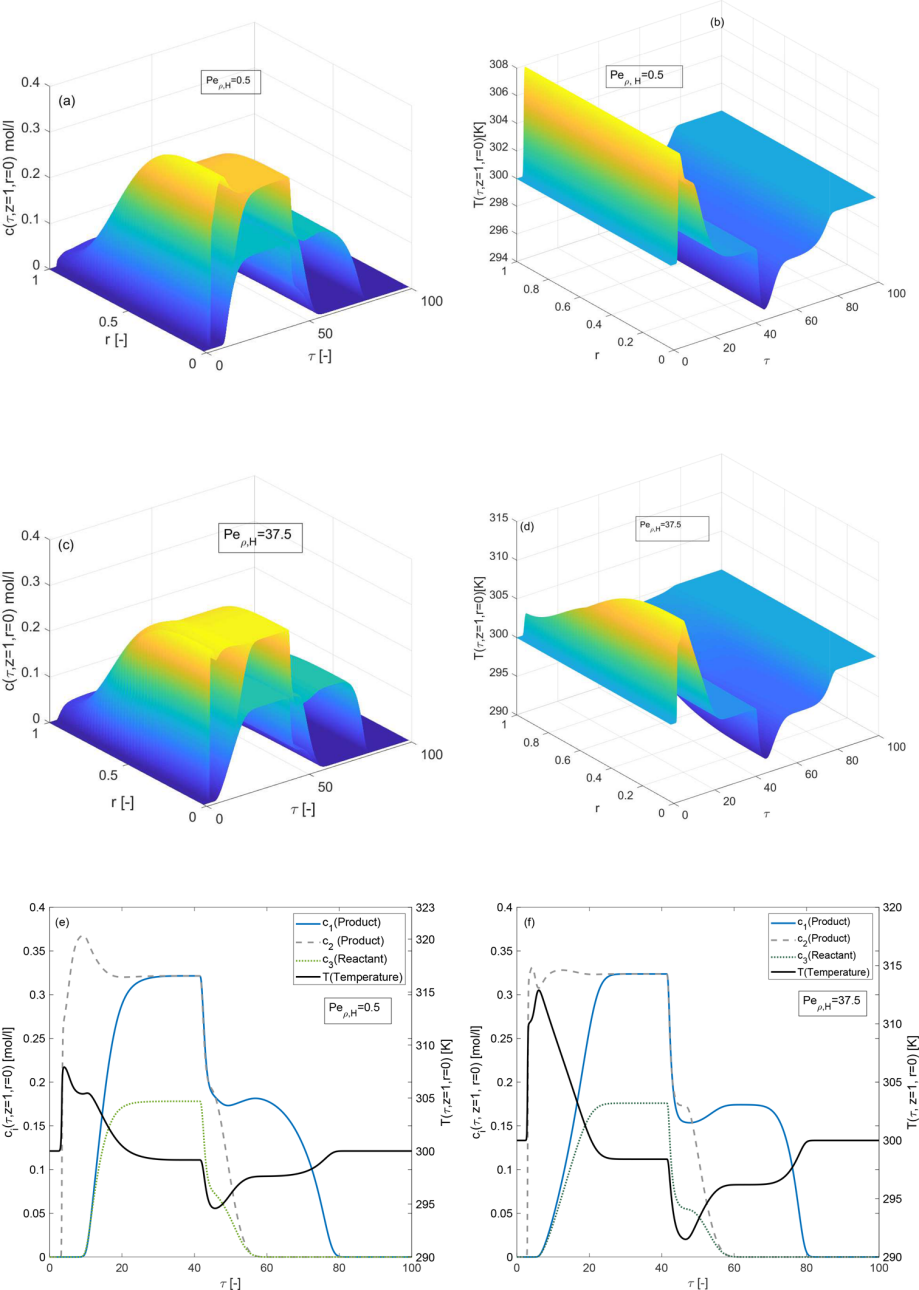
Effects of radial energy dispersion coefficient on concentration and temperature profiles. (a,b) *Pe*_ρ,*H*_ = 0.5, (c,d) *Pe*_ρ,*H*_ = 37.5, (e,f) *Pe*_ρ, *H*_ = 0.5, and Δ*H*_*R*_ = −60 kJ/mol, Δ*H*_*A*,*i*_ = −80 kJ/mol, *E*_Act_ = 60 kJ/mol, *k* = 100 min^–1^, *b*^ref^_i,I_ = 1, *b*^ref^_i,II_ = 2 for *i* = 1, 2, 3.

The effects of radial heat dispersion coefficient λ_ρ,*i*_ which is nested in the dimensionless number  is discussed in Figure 13. It can be observed from Figure 13 that only the temperature profile is affected radially by *Pe*_ρ,*H*_. The radial variations in the temperature profile are prominent for *Pe*_ρ,*H*_ = 37.5, while no radial changes appear for the smaller value of *Pe*_ρ,*H*_. The 3D plots of concentration profile show radial variations in all values of *Pe*_ρ,*H*_. In addition, the peak height of the temperature profile is high for *Pe*_ρ,*H*_ = 37.5, while the conversion rate is almost identical for both values of *Pe*_ρ,*H*_ = 0.5 and *Pe*_ρ,*H*_ = 37.5.

### Effects of injected temperature (*T*^inj^ ≠ T^ref^)

**Figure 14:**
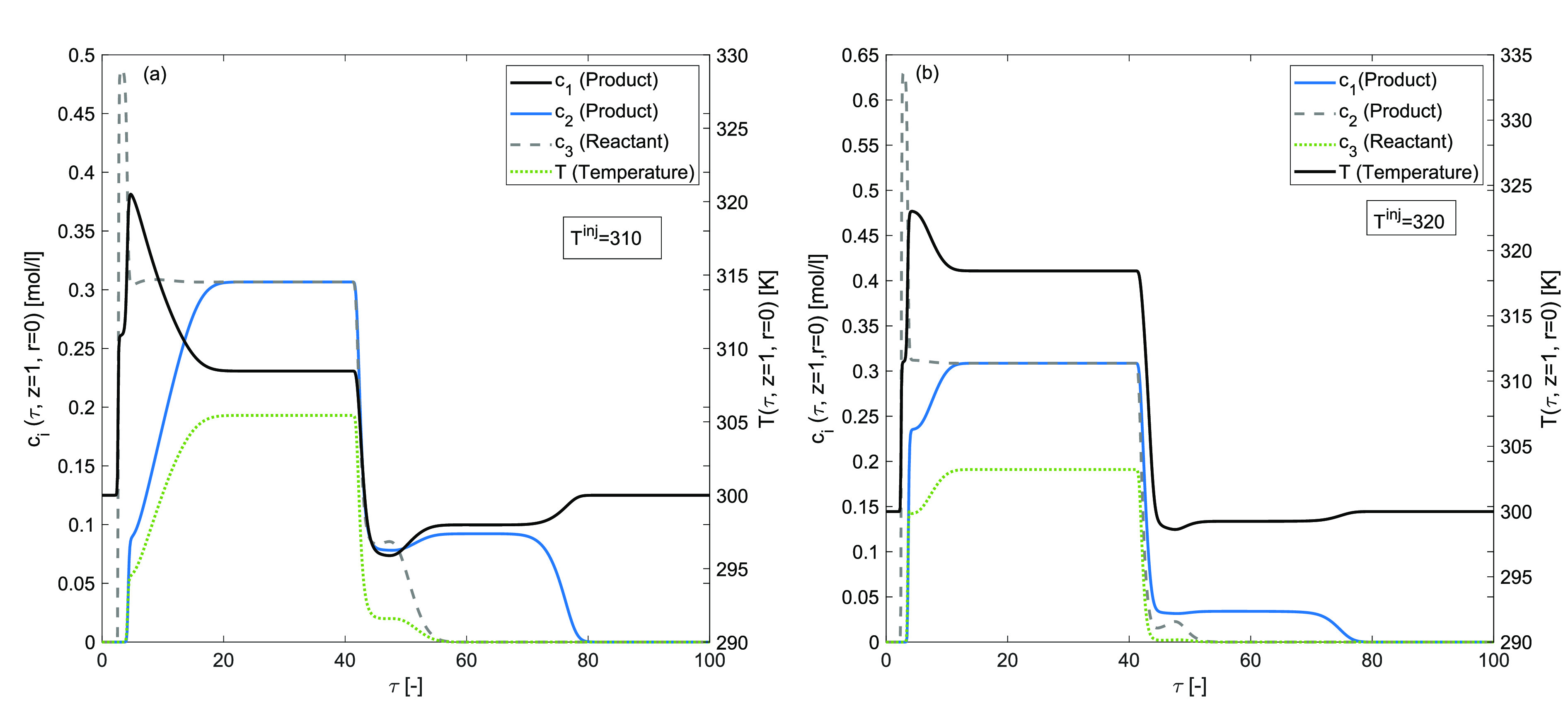
Effects of hot injection on concentration and temperature profiles. (a) *T*^inj^ = 310 K, (b) *T*^inj^ = 320 K, and Δ*H*_*R*_ = −60 kJ/mol, Δ*H*_*A*,*i*_ = −80 kJ/mol, *E*_Act_ = 60 kJ/mol, *k* = 100 min^–1^, *b*^ref^_i,I_ = 1, *b*^ref^_i,II_ = 2 for *i* = 1, 2, 3.

**Figure 15:**
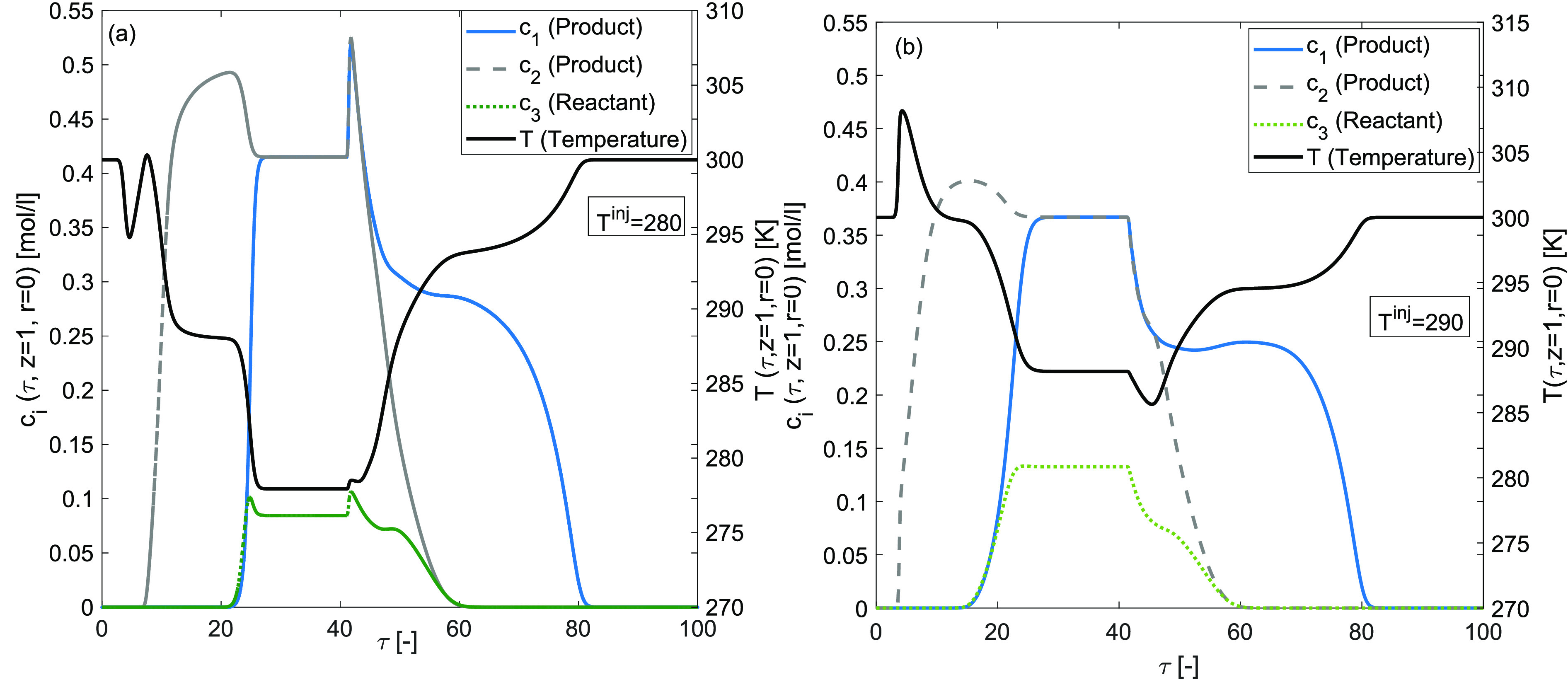
Effects of cold injection on concentration and temperature profiles. (a) *T*^inj^ = 280 K, (b) *T*^inj^ = 290 K and Δ*H*_*R*_ = −60 kJ/mol, Δ*H*_*A*,*i*_ = −80 kJ/mol, *E*_Act_ = 60 kJ/mol, *k* = 100 min^–1^, *b*^ref^_i,I_ = 1, *b*^ref^_i,II_ = 2 for *i* = 1, 2, 3.

Figures 14 and 15 display the results of the scenario when the temperature of the injected sample, *T*^inj^, is not the same as that of the mobile phase temperature, *T*^ref^. Figure 14(a,b) presents the effects of hot injection for *T*^inj^ = 310 *K* and *T*^inj^ = 320 K, while Figure 15(a,b) demonstrates the effects of cold injection for *T*^inj^ = 280 K and *T*^inj^ = 290 K, respectively. The simulations are performed for fixed values of enthalpy of adsorption Δ*H*_*A*,*i*_ = −80 kJ/mol and the enthalpy of reaction Δ*H*_*R*_ = −60 kJ/mol. A significant difference can be observed in concentration and temperature profiles’ shapes and peak heights. An increment in the temperature of the injected sample has improved the adsorption peak and reduced the desorption peak of the temperature. Analogously, a decrement in the temperature of the injected sample has curtailed the adsorption peak and enlarged the desorption peak of temperature. It is worth mentioning here that under the considered nonisothermal, Bi-Langmuir adsorption conditions and the nonlinear reaction rates, the conversion rate is maximum up to 86[%] in the case when *T*^inj^ = 280 K. The outcomes of these numerical experiments indicate that the 2D-RLKM model and the considered numerical solution technique have the potential to further develop this previously unconsidered potential of improving specific physical parameters for the enhancement of reactor performance.

## Conclusion

This work was focused on the theoretical study of reactive liquid chromatography processes in thermally insulated cylindrical columns functioning under nonisothermal and nonlinear adsorption conditions. A 2D-RLKM was formulated and numerically approximated by an efficient and accurate HR-FVM to simulate a multicomponent reactive liquid chromatographic process. The ultimate objective of this research was to analyze the impact of temperature on the efficiency of fixed bed chromatographic reactors under various adsorption conditions. The 2D-RLKM was examined for a reaction of type *c*_3_ ⇋ *c*_1_ + *c*_2_ with Bi-Langmuir equilibrium isotherms using Danckwert BCs. This isotherm was derived theoretically by assuming that the surface of the adsorbent is covered by two completely independent groups of adsorption sites. To examine the effects of various thermodynamic and kinetic parameters, numerous physically significant case studies were examined. The present 2D-RLKM model and numerical results obtained demonstrate the significant coupling of concentration and temperature fronts and identify the impact of reaction kinetics, enthalpies of adsorption and reaction, radial mass, and heat transfer coefficient, as well as different temperatures for the injected feed and the mobile phase in the process performance. It was observed that the ratio *c*_*e*_/*c*_*f*_ plays an essential role in delineating the retention time and the propagation velocity of three concentration and temperature fronts inside the reactor. Such simulations are beneficial for researchers dealing with concentrated or large volume samples in reactive liquid chromatography procedures to understand complex front propagation phenomena, optimize conditions for conducting experiments, and for improving the physicochemical parameters of the reactive units. The designed model and schemes can be used to simulate reactive chromatographic processes involving thermally insulated walls of the column, slow rates of adsorption–desorption kinetics, multicolumns, inhomogeneous packing materials, and periodic operations.

## Appendix I

### Derivation of Numerical Scheme (HR-FVM)

This study extends the semidiscrete flux-limiting, high-resolution finite volume method (HR-FVM) proposed by Koren^65^ to approximate the non-isothermal 2D-RLKM given by eqs 10–13. The discretization details for the implementation of HR-FVM on eqs 10–13 are presented as follows:

#### Spatial Discretization

It is assumed that the number of nodes along the space variables *z* and *r* is  and , respectively. Consider the following 2D-domain: [0, 1] × [0, 1], which is cloistered by the cells

1-1

The individual nodes in each cell and the constant step sizes are defined as

1-2

1-3

The initial vertex in the above equations is represented as (*z*_(1/2)_, *r*_(1/2)_) = (0, 0).

#### Cell averages

At a given time τ, introducing the average cell values of the control volume as :

1-4

where .

#### Integral form of the Nonisothermal 2D-RLKM

Integration of eqs 10–13 over Ψ_*wl*_ yields

1-5

1-6

1-7

1-8

Several different methods are available to calculate the convective fluxes at each cell interface in eqs 1-5–1-8. Here, the first-order approximation and the second-order approximation are illustrated.

#### First-Order Scheme

An approximation of first order accuracy can be acquired by evaluating the convective fluxes for each cell interface using the backward difference scheme as discussed below:^31,58,59^

1-9

1-10

1-11

1-12

#### Second-Order HR-FVM

The following flux-limiting formula is used to derive a high-resolution second-order accurate numerical scheme to approximate concentration and temperature fluxes at each cell interface:^65,69^

1-13

1-14

1-15

1-16

Here, concentration gradient ratios Θ_*i*,*w*+(1/2),*l*_, Θ_*i*,*w*–(1/2),*l*_, Γ_*i*,*w*+(1/2),*l*_ and Γ_*i*,*w*–(1/2),*l*_ for sequential fluxes are expressed as

1-17

1-18

In addition, to avoid division through zero, we require α = 10^–10^. The following flux limiting functions Φ_1_ and Φ_2_ are utilized to keep the numerical schemes’ local monotonicity.^65,70^

1-19

1-20

1-21

1-22

#### Evaluation of Differential Terms

The axial derivatives of concentration for mass and energy at each cell interface in eqs 1-5 and (1-6) are computed using a forward difference scheme:

1-23

1-24

Moreover, the following procedure is utilized to compute the radial differential terms in eqs 1-5 and 1-6.

1-25

1-26

1-27

1-28

#### Scheme Strategy at the Boundary of Domain

The flux-limiting formulations given in eqs 1-13–1-16) can not be applicable at the boundaries of domain. In order to evaluate cell-interface concentration fluxes on the boundary cells, we have employed the first-order backward scheme (c.f., eqs 1-9 and 1-12). However, the fluxes at the boundaries of the inner cells of the domain can be calculated by implementing the HR-FVM mentioned in eqs 1-13 and 1-16.

#### The Solver for ODEs

The discretization of space coordinates converts the given PDEs into an evolving system of ODEs. This system of ODEs (c.f., eqs 1-5 and 1-8) can be solved with a total variation diminishing second-order accurate Runge–Kutta (TVD-RK) scheme.^71,72^ Let  for  represent the right-hand side of eq 1-5–1-8, the TVD-RK method could then be used to upgrade  on the next two-steps as follows:

1-29

1-30

Here,  denotes the solution at present time step τ_*j*_ and  is the updated solution at the future time step τ_*j*+1_. For the stability of the numerical scheme, the time step Δ*τ* is calculated in accordance with the following Courant-Friedrichs-Lewy (CFL) condition.

1-31
